# Development of Brainstem-Evoked Responses in Congenital Auditory Deprivation

**DOI:** 10.1155/2012/182767

**Published:** 2012-06-25

**Authors:** J. Tillein, S. Heid, E. Lang, R. Hartmann, A. Kral

**Affiliations:** ^1^Department of Experimental Otology, Institute of Audioneurotechnology, Medical University Hannover, Feodor-Lynen-Strasse 35, 30625 Hannover, Germany; ^2^Institute of Sensory Physiology and Neurophysiology, J.W. Goethe University, Theodor-Stern-Kai 7, 60325 Frankfurt am Main, Germany; ^3^MedEl Company, Fürstenweg 77a, 6020 Innsbruck, Austria

## Abstract

To compare the development of the auditory system in hearing and completely acoustically deprived animals, naive congenitally deaf white cats (CDCs) and hearing controls (HCs) were investigated at different developmental stages from birth till adulthood. The CDCs had no hearing experience before the acute experiment. In both groups of animals, responses to cochlear implant stimulation were acutely assessed. Electrically evoked auditory brainstem responses (E-ABRs) were recorded with monopolar stimulation at different current levels. CDCs demonstrated extensive development of E-ABRs, from first signs of responses at postnatal (p.n.) day 3 through appearance of all waves of brainstem response at day 8 p.n. to mature responses around day 90 p.n.. Wave I of E-ABRs could not be distinguished from the artifact in majority of CDCs, whereas in HCs, it was clearly separated from the stimulus artifact. Waves II, III, and IV demonstrated higher thresholds in CDCs, whereas this difference was not found for wave V. Amplitudes of wave III were significantly higher in HCs, whereas wave V amplitudes were significantly higher in CDCs. No differences in latencies were observed between the animal groups. These data demonstrate significant postnatal subcortical development in absence of hearing, and also divergent effects of deafness on early waves II–IV and wave V of the E-ABR.

## 1. Introduction

The auditory system demonstrates extensive developmental changes during postnatal life, both in humans as well as in altricial animals (review in [1]). Which developmental effects are caused by experience and which are preprogrammed by genetic makeup (independent of hearing experience; comp. [2, 3]) is not always straightforward.

An interesting model in resolving this question is the congenitally deaf cat (CDCs, [4]). CDCs show a cochlear degeneration before hearing onset that prevents hearing experience. However, the spiral ganglion cells are well preserved [5]. This is a substantial advantage in comparison to pharmacological deafening, where the numbers of surviving spiral ganglion cells decrease within short time down to less than 50%, in some down to 10% of the counts in hearing counterparts [6]. Loss of spiral ganglion cells may lead to a down-regulation in supply of trophic factors to the cochlear nucleus (denervation effects). Loss of spiral ganglion cells is also a confounding factor for developmental studies, further aggravated by the interindividual variability in the ganglion cell loss. Electrical stimulation of the auditory nerve may thus yield a different extent of activation in the central auditory system in deafened animals. In contrast, in CDCs it is possible to test the functionality of the auditory system by electrical stimulation of the auditory nerve in a more reproducible way.

Extensive changes in the auditory cortex have been demonstrated in adult CDCs [7] as well as in neonatally-deafened cats [8]. Many of these changes are the consequence of an altered postnatal developmental sequence [9]. Consequently, auditory experience has a shaping influence on the functional maturation of the auditory system [10]. In addition, effects of auditory deprivation have been uncovered in the synapses and function of the brainstem [11] and midbrain [12–15]. It remains unclear how these changes contribute to the cortical-sensitive period for therapy of deafness with cochlear implants [16, 17].

The present study was designed to investigate the consequences of inborn deafness on the brainstem function. In auditorily naive animals of different ages, brainstem-evoked signals were measured in response to electrical stimulation through a cochlear implant. The responses were compared to electrically evoked brainstem responses in hearing (acutely deafened) controls. Electrically-evoked auditory brainstem responses (E-ABRs) are also used clinically in human cochlear implanted subjects to objectively assess the auditory function [18–20]. The present results can, therefore, be directly compared to the measurements in cochlear-implanted subjects. Additionally, brainstem-evoked responses include components from different structures in the brainstem [21] and can be used to compare effects of deprivation on several auditory structures at the same time.

The present data reveal that auditory deprivation does affect the brainstem and that these functional effects can be detected using E-ABRs. The data demonstrate that a significant portion of the developmental process is, however, preserved in complete deafness. Consequently, this part of development is dependent on genetic makeup. Finally, the present data show that different portions of the afferent auditory pathway are differentially sensitive to deprivation.

## 2. Materials and Methods

### 2.1. Animals

For the present study, 35 cats were investigated. Their age was in the range from birth (P0) to adult (>6 months), 17 animals were congenitally deaf and 18 were normal hearing controls. All congenitally deaf cats had no hearing experience before the final (acute) experiment. Two animals from the deaf white cat colony were investigated at 0 p.n. (right after birth), one deaf animal at day 3 p.n. (P3) and one deaf animal at P8, remaining animals were older than 21 days. Congenital deafness was, however, verified in the 4th week p.n. by the absence of acoustically evoked brainstem responses (see [5]). In younger animals, the same test was performed at the beginning of the experiment, whereas in animals < P10 the test cannot yield certainty on the potential hearing status later in life, since hearing thresholds drop below 100 dB SPL only after P10 in hearing cats [22].

### 2.2. Experimental Procedure

All animals were anaesthetized by subcutaneous application of ketamine-hydrochloride (Ketavet, Parker-Davis; 15 mg/kg b.w.) and xylazine-hydrochloride (Rompun, Bayer; 0.6 mg/kg b.w.). The pinna was removed at both sides, the tympanic membranes and the bullea were exposed. The animals were placed in a soundproof chamber and fixated in a stereotactic frame. For acoustic stimulation, an inversely driven calibrated Bruel and Kjear condenser microphone was placed in front of the tympanic membrane. For assessing hearing thresholds, condensation clicks (50 *μ*s) were used. Stimulation was performed at intensities in the range of 5–120 dB SPL, presented at a rate of 13 stimuli per second. For recording of auditory brainstem responses (ABRs), a small trephination was drilled at the vertex of the skull and a recording electrode was placed epidurally. The indifferent electrode was inserted in the muscles under the bulla at the site of stimulation. The recorded signal was amplified by 80 dB (Tektronix V122 and 5A22N), filtered (0.01 kHz–10 kHz, 6 dB/octave, Tektronix 5A22N), and averaged by a computer (100 repetitions). Animals classified as hearing had a click-evoked ABR threshold <40 dB SPL.

After determining the hearing thresholds, both bullae and round windows were opened. In hearing controls the hair cells were destroyed using a slow instillation (within 5 minutes) of 0.3 mL neomycine sulfate (25 mg/mL) into the scala tympani. This procedure was performed to avoid electrical stimulation of hair cells (electrophonic effects). After further 5 minutes, the neomycine was carefully washed out by Ringer's solution and deafness was verified by the absence of auditory-evoked brainstem responses (condensation click, 120 dB SPL). The deafening procedure was performed on both ears. Thereafter, a custom-made cochlear implant with five intrascalar gold contacts with a spacing of 1 mm [9] was placed in the scala tympani. The insertion depth was ~6 mm. Electrical stimulation with biphasic, charge-balanced pulses (200 *μ*s/phase, monopolar stimulation with the apicalmost electrode of the implant and an indifferent electrode placed in the muscles at the neck) was performed using an optically isolated current source. Presentation rate was 13 stimuli per second in all animals. Electrically evoked brainstem responses were recorded at varying intensities. Stimulus levels were computed from peak-to-peak amplitudes of the pulses. The highest current level was reached when facial nerve stimulation appeared. Recordings with facial nerve stimulation resulting in muscle contractions were contaminated with muscle activity and discarded from further processing.

### 2.3. Statistics

**Figure 1:**
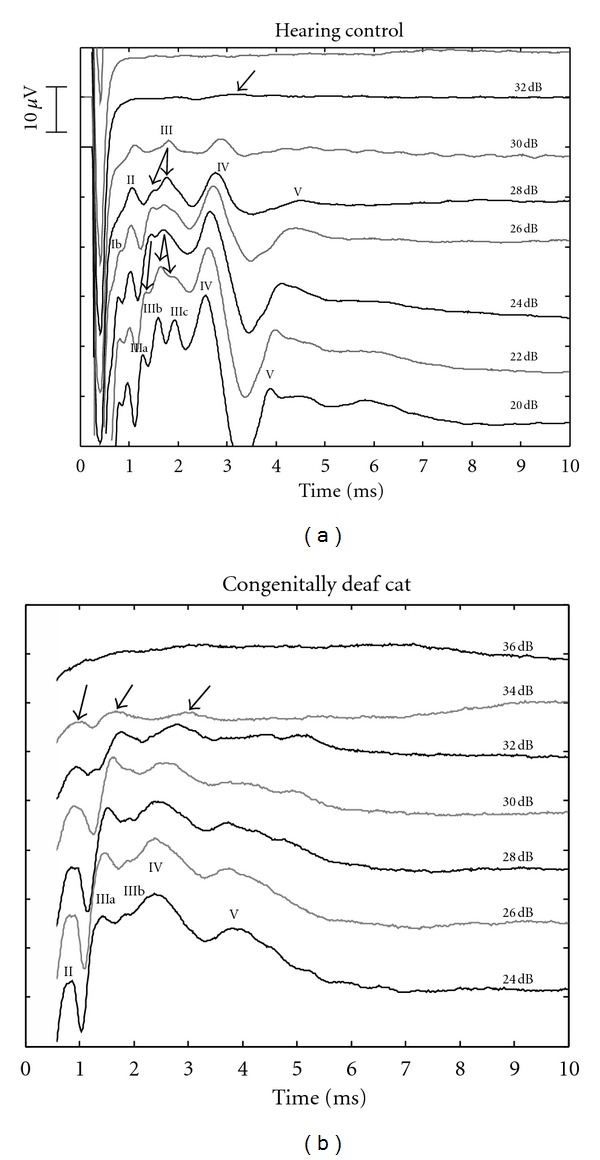
Examples of E-ABRs recorded from an adult hearing control (a) and an adult CDC (b). Arrows point to E-ABR components at threshold intensity. Current levels are given in dB attenuation (re 3 mApp). In general, similar morphology was observed, with some less-well differentiated waves in CDCs. Stimulus artifact (starting at 0 ms) was removed.

Individual waves of the electrically evoked brainstem responses (E-ABR) were designated *I*-*V* based on morphology and latency (Figure 1). Thresholds for each wave were determined as the current level at which the E-ABR curve showed a peak that appeared reproducibly at higher stimulation intensities at a similar latency. The amplitude of each wave was determined as peak-to-peak value between the maximum of each wave and the neighboring minimum of the E-ABR curve. The latency was determined as peak latency. Statistical comparisons of amplitudes and latencies were performed at 4 dB above thresholds.

Normality was tested using the Pearson-Stephens test (*α* = 10%), similarity of the distribution using the *f*-test (*α* = 10%) and final comparisons using *t*-tests (two-tailed tests *α* = 5%).

## 3. Results

In all investigated animals, the middle ear and the bulla were free of signs of infection. Deafness of all animals classified as deaf in the hearing screening (at 4 weeks p.n.) was confirmed in the acute experiment.

Electrically evoked brainstem evoked responses (E-ABRs) in adult animals revealed a morphology characteristic of acoustically evoked brainstem response (Figure 1). In most animals, 4 waves could be consistently differentiated. Additional waves appeared at high stimulus levels. To avoid misinterpretation with cortical local field potentials, the waves were numbered by Arabic numerals. Morphology of the E-ABRs was similar between deaf and hearing animals. In deaf animals, the peak of wave I was frequently hidden in the artifact of the stimulus, whereas in hearing controls it was discernible in most animals (Figure 1(a)). The waves IV and V appeared less differentiated in deaf animals (Figure 1(b)), and the variability in the morphology of E-ABRs was higher in CDCs than in controls. Wave III has split into several subcomponents at high current levels in both groups of animals. Quantitative assessment was always performed at the earliest subcomponent of wave III (wave IIIa).

### 3.1. Age Group P0–P8

None of these four animals revealed any ABRs up to click levels of 120 dB SPL, even though the ear canals have been surgically removed, the tympanic membrane was exposed and the stimulation was performed using a closed system.

**Figure 2:**
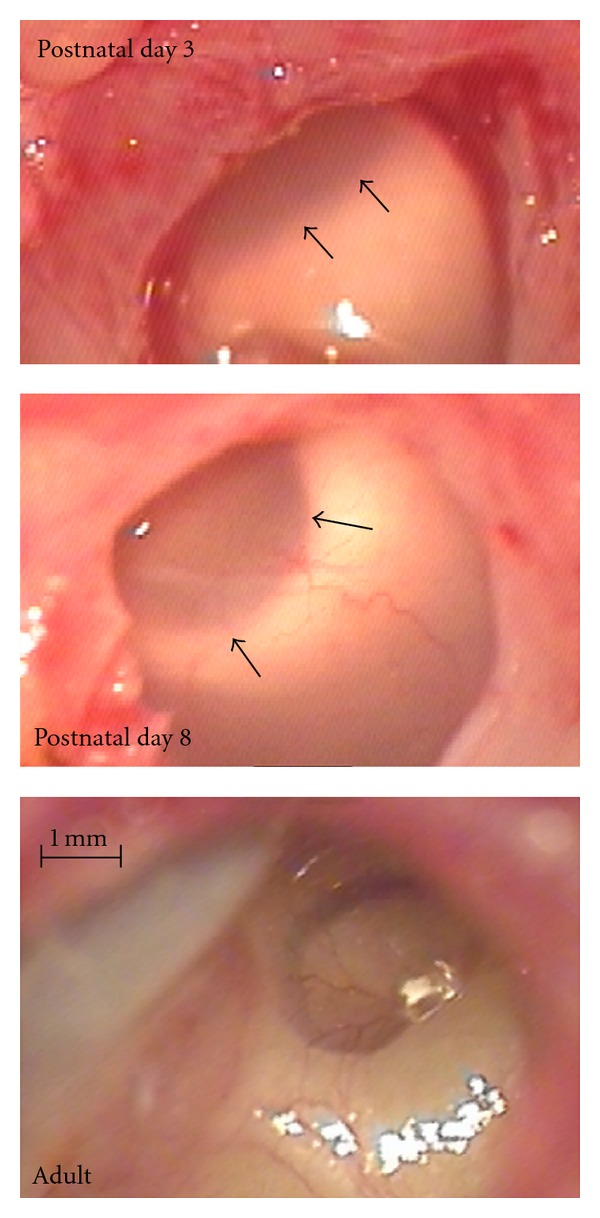
Photographs of the bulla in animals of the youngest group, compared to an adult animal. At postnatal days 0 and 3 (top), the bulla was filled with milky viscous tissue that covered the round window (its rims show through, marked by arrows). At postnatal day 8 (middle), the tissue was more translucent. In all older animals investigated, the bulla was fully pneumatized and did not show any further developmental changes (bottom).

Individual fissures of the skull were not yet ossified in this group of animals. The external meati were closed. At P3 and P8, however, they already started to open from the round widow side. Consequently, at P3 and P8, close to the tympanic membrane, the walls of the meatus did not adhere to each other anymore and the closed portion was few millimeters distant from the tympanic membrane. The middle ear was not pneumatized in this age group. The bulla was filled with a viscous whitish tissue that did not adhere to the bulla walls nor to the round window (Figure 2). It could be easily removed to allow access to the round window for cochlear implantation. The round window membrane was not as clear as in adults. In the animal investigated at P8, the fluid was more translucent than in younger animals. At P8, the round window niche was free of the viscous fluid filling the remaining part of the bulla, that is, there was a small pneumatized space in the niche of the round window. In all older animals, the middle ear and bulla were fully pneumatized and all membranes were clear (Figure 2).

Stimulation with a cochlear implant allowed a controlled and comparable testing of animals despite a nonfunctional cochlea. Using monopolar configuration, the maximal portion of the auditory nerve was stimulated and positional effects were minimized.

**Figure 3:**
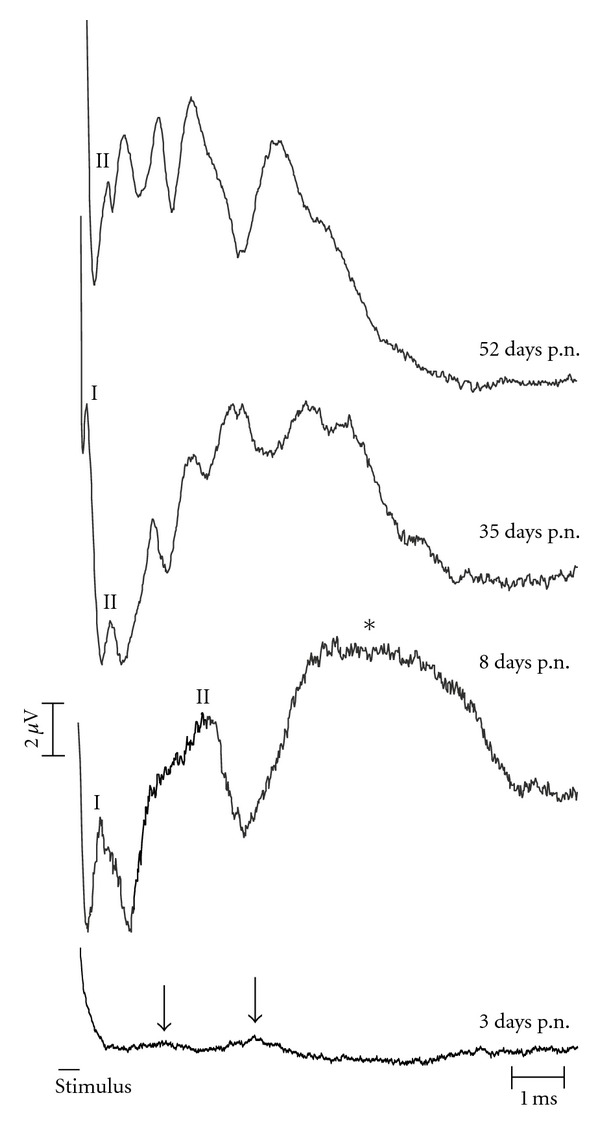
Development of E-ABRs in the first 52 days postnatally. All animals are from the white cat colony, but only after hearing onset (after day 10 p.n.) CDCs can be differentiated from white cats with residual hearing. Stimulus: biphasic pulse, 200 *μ*s/phase at 10 dB above threshold. Stimulus duration is shown as a black bar, the artifact is removed form the recordings. At day 3 p.n., even at this high stimulation intensity the amplitudes of the waves were very small and only two waves could be differentiated from noise (marked by arrows). At day 8 p.n., all waves were observed (at the high intensity shown, waves III–V fused into a single large wave marked by the asterisk).

Substantial maturation of E-ABRs was observed within the first days of life (Figure 3). At P0, E-ABR was not discernible in both investigated kitten. It was present but very small at P3 (Figure 3). Its amplitudes increased and latencies systematically decreased within the following weeks (Figure 3). Due to the atypical E-ABR morphology in very young animals, the wave assignment was not equivocal at P3.

### 3.2. Comparison of Developing Animals

The range of suprathreshold intensities that were possible to test differed in individual animals due to muscular artifacts. These were generated by facial nerve stimulation at high current levels. Consequently, the dynamic range of E-ABRs that was possible to evaluate varied between 4 and >10 dB above threshold. To include all investigated animals, individual E-ABRs waves were compared at 4 dB above thresholds. Only positive peaks of the E-ABR components were processed.

**Figure 4:**
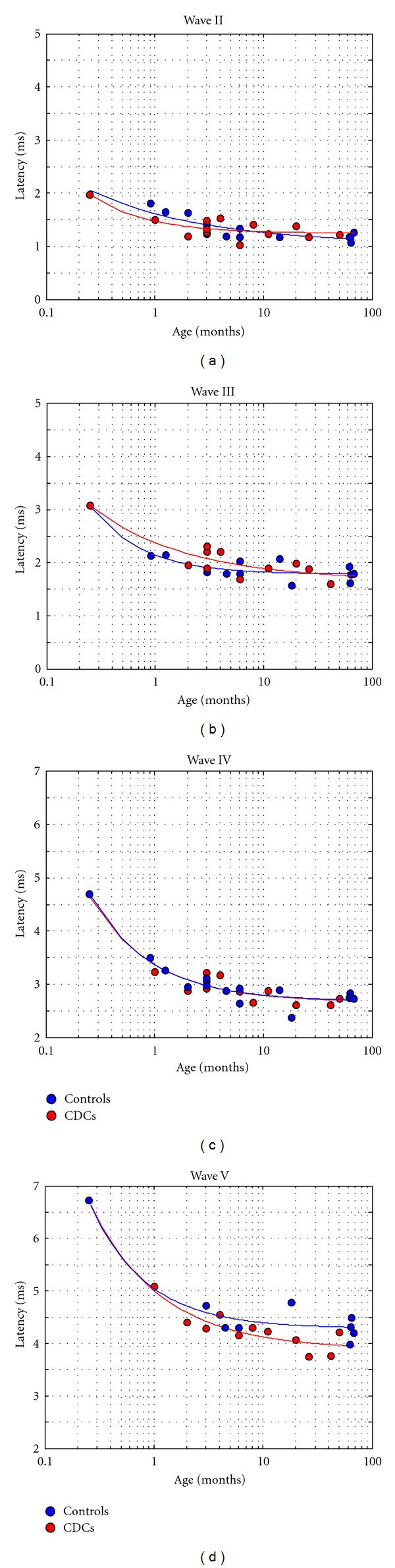
Development of wave latencies in hearing controls and CDCs, shown are peak latencies from all animals. Not in every animal all waves could be identified. The animal recorded at day 8 p.n. (before hearing onset) was considered a common starting point for the development in both groups. Systematic differences between animal groups were not observed, only in wave V had a tendency toward shorter latencies in CDCs.

With the exception of an increase in amplitudes between day 3 and 35 p.n. (Figure 3), the amplitudes showed a high degree of variability and no clear developmental trend. When analyzing the developmental pattern of latencies from postnatal day 8 on (at which for the first time wave components could unequivocally be compared in wavewise manner to adult animals), a decrease in latencies could be observed, most prominent within the first 3 months (Figure 4). For the purpose of comparing the developmental pattern, the data were pooled into two groups: from 0–3 months (comp. [23]) and above 4 months. When comparing such young and older animals within the hearing group, the latencies were significantly different for most waves of the E-ABR (wave I  *P* = 0.0489; wave II  *P* = 0.00115; wave III  *P* = 0.030; wave IV  *P* = 0.012; wave V  *P* = 0.021; two-tailed *t*-test), demonstrating a developmental change. In deaf animals, the developmental change was similar, whereas for wave II, the differences did not reach the level of statistical significance (wave II  *P* = 0.084; wave III  *P* = 0.044; wave IV  *P* = 0.047; wave V  *P* = 0.036). In HCs and CDCs, the timecourse of development of brainstem evoked response latencies was similar (Figure 4).

### 3.3. Detailed Comparison of Matured Animals (>3 Months)

As no pronounced age-related changes were observed in waves II, III, and IV after the 3rd month (Figure 4), animals older than 3 months were pooled together and hearing controls were statistically compared to CDCs (8 congenitally deaf and 9 hearing controls).

### 3.4. Thresholds

**Figure 5:**
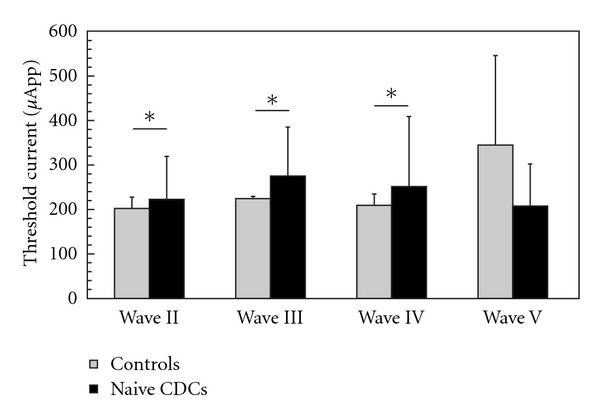
Comparisons of wave thresholds in matured animals (older than 3 months). Significantly larger thresholds were observed in CDCs for waves II, III, and IV. The trend in wave V is in opposite direction (two-tailed *t*-test at *α* = 5%).

Lowest E-ABR thresholds did not significantly differ between hearing and deaf animals if all waves were included (186 ± 52.6 *μ*A in controls versus 180.8 ± 61.8 *μ*A in deaf). Already at threshold intensity, E-ABRs showed most frequently waves III/IV and V. However, in some animals, also (sometime only) earlier waves (typically II) or later waves (typically IV) appeared at threshold. To compare the E-ABRs in a wave-specific manner, the thresholds were also compared on the basis of “wave thresholds” (Figure 5). For wave V, the thresholds did not differ between deaf and hearing animals (345 ± 201 versus 209 ± 94 *μ*A,  *P* = 0.13; Figure 5). On the other hand, the thresholds of waves II, III, and IV were significantly higher in CDCs (II: 202 ± 26 *μ*A in controls versus 223 ± 97 *μ*A in deaf,  *P* = 0.03, two-tailed *t*-test; III: 225 ± 15 *μ*A in controls versus 275 ± 110 *μ*A in deaf,  *P* = 0.05, two-tailed *t*-test; IV: 210 ± 26 *μ*A versus 251 ± 94 *μ*A,  *P* = 0.03, two-tailed *t*-test). This result indicates that potentially divergent deprivation effects take place in the generators of the early waves II–IV and the wave V. Additionally, deaf cats consistently demonstrated higher interindividual variance in thresholds.

### 3.5. Amplitudes

**Figure 6:**
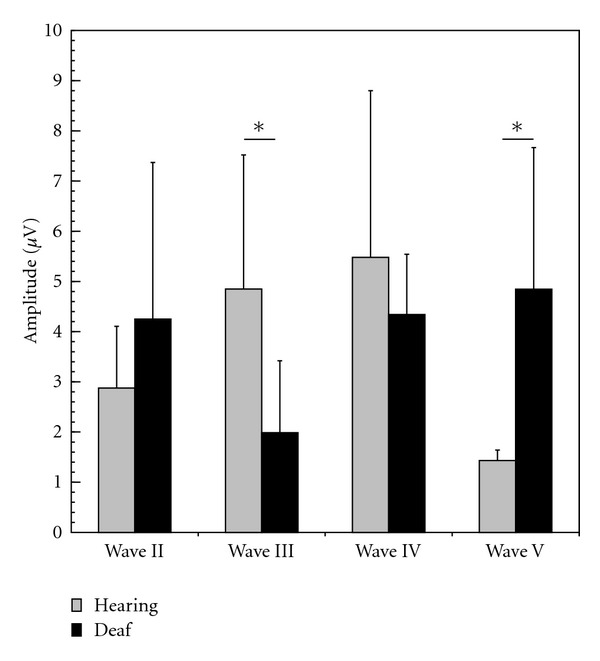
Wave mean peak amplitudes in matured animals (older than 3 months). In CDCs, wave III had lower and wave V higher amplitude (two-tailed *t*-test at *α* = 5%).

Wave amplitudes were typically below 20 *μ*V and showed some interindividual variability (Figure 6). When comparing deaf and hearing animals at 4 dB above threshold, significant differences were observed for wave III (4.85 ± 2.7 *μ*V in controls versus 1.99 ± 1.43 *μ*V in deaf;  *P* = 0.03, two-tailed *t*-test) and wave V (1.43 ± 1.18 in controls versus 4.84 ± 2.82 in deaf;  *P* = 0.04, two-tailed *t*-test). Also in this respect, a discrepant effect of deprivation was observed on wave III (smaller in CDCs) and wave V (larger in CDCs).

### 3.6. Latencies

**Figure 7:**
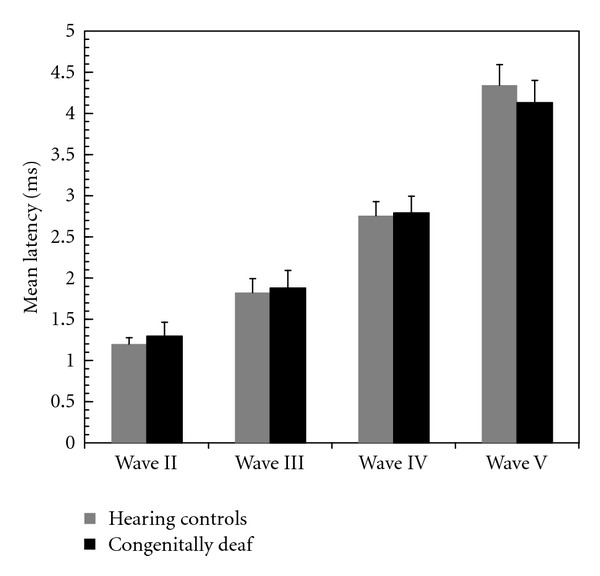
Mean peak latencies of all waves in matured animals (older than 3 months). No statistically significant differences were observed between CDCs and hearing controls.

Wave I was difficult to evaluate in deaf cats, as the recordings did not include this peak in sufficient number of animals. This was attributed to a shorter latency of this wave in deaf animals (by which the wave and the stimulus artifact coincided). Latencies of waves II–V at 4 dB above threshold were not significantly different between deaf and control animals in this age group (Figure 7). Also all interpeak latencies were not significantly different (*P* = 0.9 for interpeak intervals II-III,  *P* = 0.86 for interpeak III–IV,  *P* = 0.17 for interpeak IV-V,  *P* = 0.14 for interpeak II–V).

## 4. Discussion

For the first time the present manuscript describes development of brainstem-evoked responses in inborn deafness. The developmental pattern of brainstem evoked responses with well-controlled auditory stimulation (through a cochlear implant) was compared between hearing controls and congenitally deaf cats. Using electrical stimulation it has been possible to investigate brainstem-evoked response development prior to hearing onset. At postnatal day 3, E-ABRs could be evoked, corresponding to “acoustic” studies with first responses at postnatal day 2-3 using an unphysiologically high sound pressure level of 140 dB [24].

The study demonstrates a developmental change in the brainstem evoked responses in both hearing and deaf animals. Thus, many functional developmental steps in the brainstem are set-in by genetic programs and do not require a functional cochlea. This stands in contrast to the findings in the auditory cortex, where the developmental pattern has been found extensively modified by deafness [7].

However, several effects of deprivation were observed in the brainstem responses. Qualitatively, the E-ABR appeared more smeared and were more variable in CDCs and indicated a desynchronization of the underlying neuronal activity in CDCs. Quantitatively, there was an increase in thresholds of early E-ABR waves and divergent effects of deafness on waves II, III, and V with respect to amplitudes and thresholds. Wave V has generators in the midbrain, whereas earlier waves are generated in the brainstem [21, 25, 26]. These results speak for a different response to deprivation in more peripheral and more central parts of the auditory pathway. Potentially, the wave V effects (higher amplitude and lower threshold in deaf animals) correspond to compensation of the missing input by downregulated inhibition and upregulated excitatory transmission, as described in the inferior colliculus [27] and auditory cortex of deaf or deafened animals [9, 28].

Considering that brainstem-evoked responses are not capable reflecting all details of the differences in synaptic and neuronal function between HCs and CDCs, the effects of deafness on waves II–IV are well in agreement with studies demonstrating synaptic reorganization in the cochlear nucleus following congenital deafness [11, 29–35], as well as in the more central parts of the auditory system (reviewed in [7]).

### 4.1. Methodological Discussion

Several methodological factors could have influenced the present results. First of all, amplitudes of brainstem responses are known to have a higher variability than latencies (reviewed in [36]). This is the consequence of the fast temporal order of the components, causing them to partly overlay. In acoustic stimulation, some authors filtered out the low-frequency component to facilitate the quantification [37]. We decided against this procedure due to the presence of the electrical stimulus artifact that complicated the offline filtering. Nonetheless, statistically significant results were obtained also for amplitudes. Therefore, we considered our amplitude quantification as sufficiently robust.

The threshold of E-ABRs depends on the position of the cochlear implant within the scala tympani. Positioning the stimulation electrodes closer to the modiolus can decrease E-ABR thresholds by 6 dB [38]. The position of the electrode within the scala tympani cannot be controlled by the surgeon and could act as a confounding factor. Nonetheless, a systematic bias between the investigated age groups is highly unlikely, given that the lowest E-ABR threshold was the same in both groups. The effect of electrode position was further minimized by using a monopolar stimulation configuration.

The number of surviving spiral ganglion cells may significantly influence threshold currents [39]. Nonetheless, in contrast to neonatally deafened animals, the congenitally deaf cats do not suffer from a pronounced degeneration of the spiral ganglion cells in the basalmost halfturn of the cochlea and the ages investigated here [5, 6]. It is the basalmost halfturn of the cat cochlea where a cochlear implant can be inserted [40]. Therefore, a systematic effect of the spiral ganglion cell loss on the present results is not probable.

Finally, the repetition rate could have influenced the results, especially in the youngest animals. To avoid confounding the results by different repetition rates in different age groups, we decided to select a constant low repetition rate (13 Hz), whereas the brainstem response in adults can be reliably recorded up to the repetition rates of 70 Hz [41]. Even in animals at P3, we could record a brainstem evoked response. In consequence, the selected rate appears to be a good compromise. Cortical responses at P8 have latencies of ~50 ms [7], thus this repetition rate (with interstimulus intervals of ~77 ms) should allow a sufficient activation of the entire afferent auditory system. Nonetheless, the repetition rate needs to be taken into consideration as a factor lowering the amplitudes of E-ABRs in youngest animals (day 0, 3 and 8 p.n.).

### 4.2. E-ABR Generators

Electrically evoked brainstem responses are similar to acoustically evoked brainstem responses [42–45]. Consequently, one can assume the same generators of both types of signals. Here, we used a terminology of brainstem response waves that rests on the terminology used with acoustic stimulation of the cat auditory system. We assume that the waves found in electrically evoked brainstem responses correspond to the ones in acoustically evoked brainstem responses, as the morphology and the latency range (considering the absence of acoustic transduction in the inner ear) correspond well. Interestingly, in hearing controls wave III reproducibly broke up into 3 waves with increasing electrical intensity. Similarly, with high-intensity acoustic stimulation, wave III sometimes splits into 2 components [37]. This finding supports the theory that wave III is caused by activation of up to 3 different structures in the brainstem [21], and that acoustical and electrical stimulation result in ABRs with similar generators and morphology of responses. Possibly, the “hypersynchronization” of the electrically evoked activity compared to acoustic stimulation [46] additionally allows to better reveal these tree generators. Also, in CDCs, a similar effect on wave III has been observed, although wave III broke up only into 2 well-differentiated components. The observation of fewer subcomponents of wave III in CDCs further supports a desynchronization of the underlying neuronal activity in deafness. All shown quantitative comparisons were consequently performed on the component of wave III that appeared with shortest latency (wave IIIa).

### 4.3. Development of Auditory Pathway

Development of brainstem responses to electrical stimulation has not been systematically investigated yet. On the other hand, development of brainstem-evoked responses to acoustical stimulation has been investigated in great detail in hearing cats (comp. [23, 24, 47, 48]). When latencies are plotted as a function of age at same sensation levels in these acoustically stimulated animals, the data correspond well to the present study: also there the main development took place in the first 90 days [23]. In hearing, acoustically stimulated animals, exponential decays similar to the present study have been observed (ibid., comp. Figure 4). The electrically evoked responses in the present study had slightly shorter latencies when compared to the acoustical of the previous studies, explicable by by-passing the hair cell-primary afferent synapse with cochlear implant stimulation.

The present experiments provide further support for the ABR hearing screening procedure performed on the deaf white cats [5]: none of the animals classified as deaf showed any signs of hearing in the acute experiments. Additionally, even though electrically evoked responses could be demonstrated at P3, no signs of hearing were observed in the animals tested between P3 and P8, even after surgical opening of the closed outer ear canal. Additionally, performing the E-ABRs in the same animals, we confirmed that the central auditory system beyond the cochlea was functional. Consequently, absence of acoustical responses was solely due to lack of cochlear function. From P3 on, morphological degeneration of the organ of Corti in CDCs is quickly progressing, with loss of cochlear microphonics and hair cells [4, 49].

Electrically evoked brainstem responses can be recorded even before acoustically evoked responses with physiological sound pressure levels. This demonstrates that the afferent auditory pathway (at least in part) is already functional before significant hearing is possible. Additionally, it shows that the general pattern in the afferent auditory pathway develops in absence of auditory experience (see also [5, 45, 50]).

### 4.4. Wave I in CDCs

Wave I corresponds to compound action potentials of the auditory nerve [51]. In the present experiments, wave I was not consistently observed in CDCs. Thus, one can speculate that the action potentials in the auditory nerve were generated less synchronously in CDCs, possibly at a more central site when compared to controls. The reason for such a shift in action potential generation site could be a change in the physiological properties of primary afferents in deaf cats, possibly by demyelination of the most peripheral portions of the primary afferents [52, 53]. As the highest probability for action potential generation is at the nodes of Ranvier, and the demyelination is connected with changed distribution of fast voltage-sensitive sodium channels along the primary afferents, the demyelination could lead to a shift of the action potential generation site to more central nodes of Ranvier ([52, 53]; compare also discussion in [40]). That would lead to shorter wave I latencies. However, the absence of wave I in the present experiments could have also been caused by a higher asynchrony of action potential generation in the auditory nerve by differentially shifting the action potential generation site along the auditory nerve in different fibers. The later waves did not show a systematic latency difference between controls and CDCs, favoring the asynchrony hypothesis or indicating that some additional deprivation-induced delay has to take place in CDCs between wave I and wave II.

### 4.5. Matured Animals

The present results on matured animals are in accordance with data on neonatally pharmacologically deafened adult cats. Pharmacologically deafened adult animals show smaller E-ABR amplitudes [31]: this was also the case in the present animals, although it was only statistically significant for wave III. A correlation between the amplitudes of E-ABRs and the number of spiral ganglion cells was demonstrated [51], although for later waves of the E-ABR, this correlation was weaker [42, 54, 55]. Nonetheless, significant correlation of wave IV amplitude with spiral ganglion cells has also been shown [56]. A more pronounced denervation effects on the development or degeneration of the brainstem in the neonatally deafened cats as a consequence of the loss of spiral ganglion cells early in postnatal life could be one reason why the difference in amplitudes was more pronounced in adult neonatally deafened cats when compared to CDCs from the present study.

Another mechanism for smaller amplitudes of the E-ABRs in deaf cats could be a loss of neurons in the cochlear nucleus of deprived animals. This was shown in rodents when the neuronal activity was completely silenced before hearing onset [57, 58]. To date, there is no systematic study on the functional properties of “deaf” auditory nerve fibers in CDCs. A report on six animals from a deaf white cat colony with some residual hearing showed severely reduced spontaneous activity in auditory nerve fibers (assessed with intracellular recordings) of animals with high hearing thresholds [59]. However, despite a reduction in the total nuclear volume within the auditory brainstem [29–31], no evidence for a decrease in neuronal counts has been reported, neither for CDCs nor for neonatally deafened cats [5, 31].

Numerous studies described morphological changes in the synapses of the brainstem in CDCs [11, 32–35]. It is probable that the decrease in amplitude of wave III is due to weakening of synaptic activity and desynchronization rather than anatomical loss of central auditory neurons.

Delayed or incomplete myelination in CDCs may be a further reason for smaller E-ABR amplitudes in CDCs. However, the absence of differences in interpeak latencies between CDCs and HCs argue against a delayed transmission along the auditory brainstem and thus also against this interpretation. Further studies are required to eventually identify the mechanism behind smaller E-ABR amplitudes in CDCs.

There was an increase in individual wave II–IV thresholds in CDCs. This was also observed in adult neonatally deafened animals [31]. Increase in thresholds of individual E-ABR waves can be explained by weakening of synaptic efficacies along the auditory pathway [11, 32, 34, 35, 60] or by desynchronization of their activity. Deafness-induced increase in jitter of action potentials in inferior colliculus has been shown in neonatally deafened animals [15]. Nonetheless, the individual contribution of these mechanisms cannot be assessed solely from brainstem-evoked responses.

### 4.6. Human ABRs

Development of human brainstem-evoked responses has been also described in great detail in hearing children (review in [36]). Brainstem-evoked responses represent a valuable tool for exploring the function of the afferent auditory pathway in deafness, with remarkably corresponding data between cochlear-implanted humans and animals. Brainstem responses of cats and humans, after appropriate normalization, show similar developmental rates for the early waves, whereas humans show a slower development for the late waves (wave V) [61]. As in the present study, also in brainstem-evoked responses of humans, a similar development has been observed in hearing children and in deaf, cochlear-implanted children [62]. In consequence, also in deaf children the brainstem-evoked responses develop comparably to hearing peers and do not require cochlear input.

Correspondingly, no sensitive periods in the development of brainstem response of humans have been observed with chronic electrical stimulation (cochlear implant use, [62, 63]). These are most likely determined by the parts of the auditory system beyond the brainstem and midbrain ([62]; comp. also [64]). The most probable candidate, strongly interacting with all levels of the auditory system, is the auditory cortex, where developmental sensitive periods have been observed both in CDCs as well as in cochlear-implanted humans (reviewed in [7]). In combination, this evidence indicates that sensitive periods for cochlear implantation [63, 65] are determined by the auditory cortex.

## 5. Conclusions

Electrically evoked brainstem responses show postnatal development even in complete absence of hearing experience. Deprivation-induced effects include reductions of wave III amplitude, increase of wave V amplitude, and increases of wave thresholds. No effects of deafness on E-ABR latencies were found. The results indicate desynchronization and/or weakening of synaptic activity in auditory brainstem and some additional compensatory “hypersensitivity” in the midbrain of deaf animals.
