# Erratum to “Development of Brainstem-Evoked Responses in Congenital Auditory Deprivation”

**DOI:** 10.1155/2012/168297

**Published:** 2012-12-23

**Authors:** J. Tillein, S. Heid, E. Lang, R. Hartmann, A. Kral

**Affiliations:** ^1^Department of Experimental Otology, Institute of Audioneurotechnology, Medical University Hannover, Feodor-Lynen-Strasse 35, 30625 Hannover, Germany; ^2^Institute of Sensory Physiology and Neurophysiology, J.W. Goethe University, Theodor-Stern-Kai 7, 60325 Frankfurt am Main, Germany; ^3^MedEl Company, Fürstenweg 77a, 6020 Innsbruck, Austria

The caption of Figure 1 in the paper at doi:10.1155/2012/182767 has to be corrected as shown here. Also, [1] should be corrected as follows: J. Tillein, S. Heid, E. Lang, R. Hartmann, and A. kral, “Development of brainstem-evoked responses in congenital auditory deprivation,” Neural Plasticity, vol. 2012, Article ID 182767, 11 Pages, 2012.

## Figures and Tables

**Figure 1:**
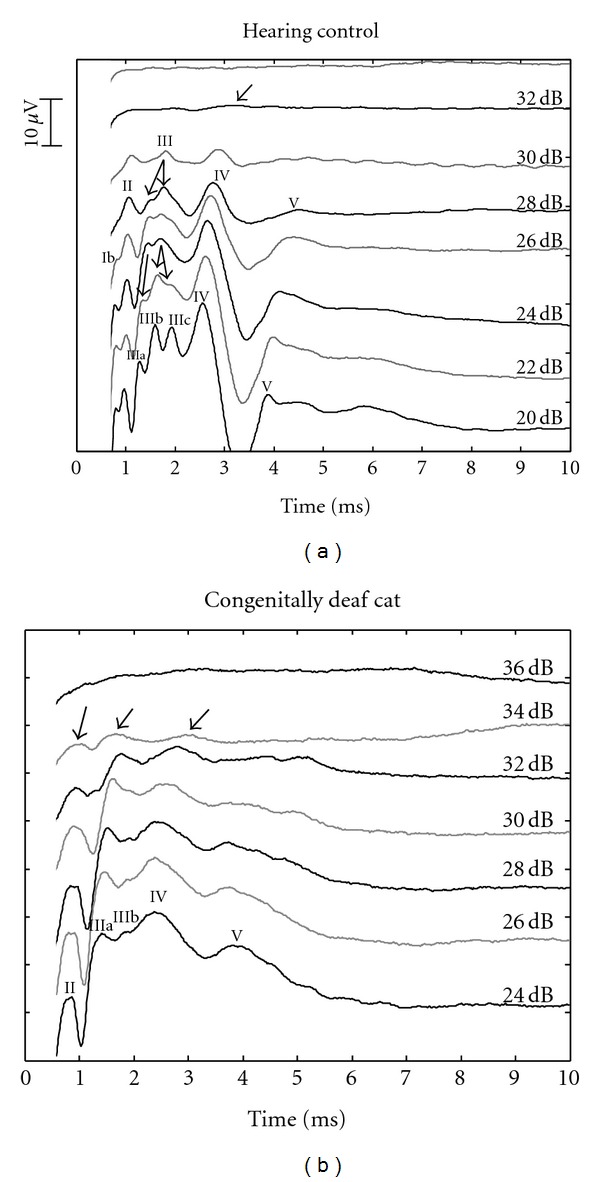
Examples of E-ABRs recorded from an adult hearing control (a) and an adult CDC (b). Arrows point to E-ABR components at threshold intensity. Current levels are given in dB attenuation (re 3mApp). In general, similar morphology was observed, with some less-well differentiated waves in CDCs. Stimulus artifact (starting at 0 ms) was removed.

